# Associations between surrogate insulin resistance indexes and osteoarthritis: NHANES 2003–2016

**DOI:** 10.1038/s41598-024-84317-z

**Published:** 2025-01-10

**Authors:** Youmian Lan, Qiongbing Zheng, Meijing Li, Jiexin Chen, Dongyang Huang, Ling Lin

**Affiliations:** 1 Department of Rheumatology and Immunology, The First Affiliated Hospital of Shantou University Medical College, Shantou, 515041 China; 2 Department of Cell Biology and Genetics, Key Laboratory of Molecular Biology in High Cancer Incidence Coastal Chaoshan Area of Guangdong Higher Education Institutes, Shantou University Medical College, Shantou, 515041 China; 3 Department of Rheumatology, Shantou University Medical College, Shantou, 515041 China; 4 Department of Neurology, Shantou Central Hospital, Shantou, 515041 China

**Keywords:** Osteoarthritis, Insulin resistance, Triglyceride glucose, TyG-WHtR, NHANES, Databases, Endocrine system and metabolic diseases, Osteoarthritis

## Abstract

Insulin resistance (IR) and abdominal obesity are key in osteoarthritis (OA) development. The triglyceride glucose (TyG) index, along with indicators such as the visceral adiposity index (VAI), and lipid accumulation product (LAP), are increasingly used to measure IR. This study aims to explore the associations between surrogate IR indexes and OA, assessing their diagnostic efficacy within American populations. This study included 14,715 adults from the National Health and Nutrition Examination Survey 2003–2016. Logistic regression models and restricted cubic spline were used to explore the relationship between surrogate IR indexes and OA. Receiver operating characteristic curves were constructed to assess the diagnostic efficacy of these indices, with the area under the curve (AUC) as the metric. TyG, glucose triglyceride-waist circumference (TyG-WC), glucose triglyceride-body mass index (TyG-BMI), glucose triglyceride-waist height ratio (TyG-WHtR), VAI and LAP were significantly and positively associated with the prevalence of OA (all *p* < 0.01). After adjusting for various potential confounders, TyG-WC, TyG-BMI, TyG-WHtR and LAP remained significantly correlated with the prevalence of OA. Furthermore, restricted cubic spline revealed a nonlinear association between TyG-BMI, TyG-WHtR and LAP (all *P*-non-linear < 0.05). Receiver operating characteristic curves indicated that TyG-WHtR (AUC 0.633) demonstrated more robust diagnostic efficacy. Additionally, the sensitivity analysis produced results consistent with the primary findings. TyG and its combination with obesity indicators and LAP, are positively associated with the prevalence of OA, with TyG-WHtR showing the highest diagnostic efficacy.

**Supplementary Information:**

The online version contains supplementary material available at 10.1038/s41598-024-84317-z.

## Introduction

Osteoarthritis (OA) is a multifaceted joint disease involving alterations in articular cartilage, subchondral bone, ligaments, capsule, synovial membrane and periarticular muscles. It is a prevalent degenerative condition in the elderly, a major cause of chronic pain and disability, and a significant burden on global public health resources^1,2^. The prevalence of this illness has significantly increased in recent decades, primarily due to the aging population and rising obesity rates^3^. It is estimated that approximately 595 million people (7.6%) worldwide had this condition in 2020, with projections suggesting a rise to 1 billion by 2050. The highest age-standardised prevalence rates are observed in the Asia-Pacific, North America, and Eastern Europe regions^2^.

Previous studies have drawn a link between OA and metabolic syndrome (MetS), which is characterized by hypertension, hyperglycemia, dyslipidemia, and abdominal obesity^4–7^. Insulin resistance (IR), a crucial component of MetS, refers to a reduced responsiveness of insulin in target tissues or organs. The hyperinsulinemic-euglycemic clamp serves as the gold standard for diagnosing IR, but its invasiveness, complexity, and cost limit its use in epidemiological studies. While the homeostasis model assessment of IR is convenient to use, it is not suitable for diagnosing patients undergoing insulin therapy^8,9^. In this context, alternative markers derived from blood glucose and lipid profiles, as well as specific anthropometric measurements, such as the visceral adiposity index (VAI), lipid accumulation product (LAP), and triglyceride-glucose index (TyG), are increasingly employed as simple and effective tools for identifying IR^10–12^. Extensive research indicates that these surrogate IR indexes are significantly associated with the prevalence of cardiovascular disease^10^, ischemic stroke^13^, and diabetes^14^. Obesity is a major contributor to OA and interacts with IR in its development. Recent studies have combined the TyG with various obesity measures to develop new indices, such as glucose triglyceride-waist circumference (TyG-WC), glucose triglyceride-body mass index (TyG-BMI), and glucose triglyceride-waist height ratio (TyG-WHtR). These new indices demonstrate superior accuracy and sensitivity in assessing metabolic disease risk compared to TyG, waist height ratio (WHtR), or body mass index (BMI) alone^15–17^.

Recent studies suggest that LAP, TyG, and TyG combined with obesity measures may predict OA^11,18,19^. However, the relationship between surrogate IR indexes and OA needs further exploration. Using data from the National Health and Nutrition Examination Survey (NHANES) 2003–2016, this study aims to investigate the associations between surrogate IR indexes and OA, evaluating their diagnostic efficacy to identify the superior indicators for assessing OA.

## Materials and methods

### Data source and study participants

**Fig. 1:**
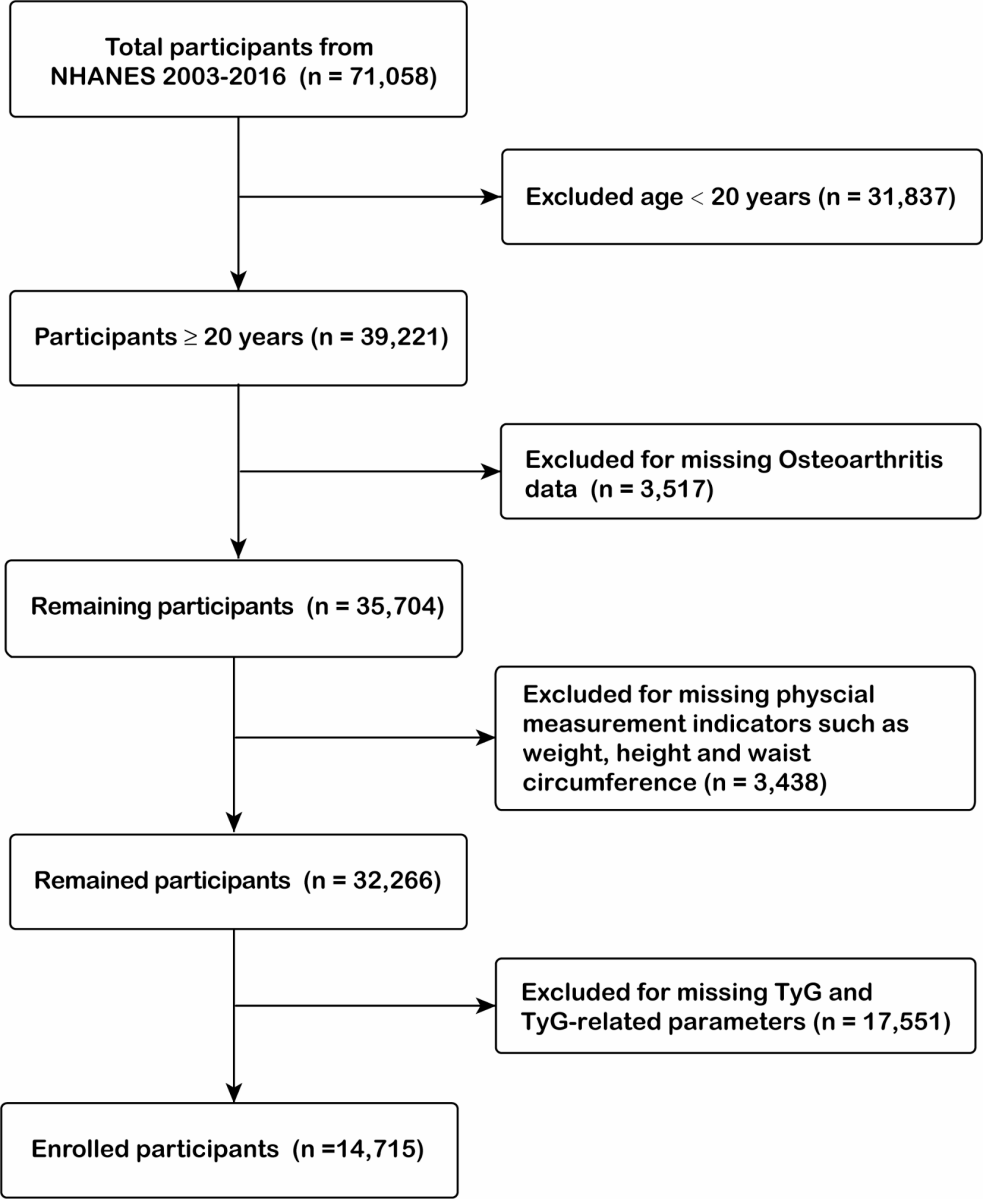
Flow chart of sample selection.

This cross-sectional study utilizes data from seven NHANES cycles (2003–2016), encompassing 71,058 participants who underwent extensive population surveys, laboratory examinations, and health survey questionnaires. To ensure accuracy and reliability, we implemented rigorous data screening and exclusion processes. Since our study focused on surrogate IR indexes in adults, participants being under 20 years old (*n* = 31,837) were excluded. We further excluded participants with missing data for OA (*n* = 3517), physical measurement indicators such as weight, height and waist circumference (WC) (*n* = 3438), and TyG and TyG-related parameters (*n* = 17,551). Finally, this study included a large national representative sample (*n* = 14,715) of the general adult American population. The flowchart of the study is shown in Fig. 1.

All participants have signed a written informed consent form, which has been approved by the Ethics Review Committee of the National Center for Health Statistics (Protocol #98-12, Protocol #2005-06, Continuation of Protocol #2005-06, Protocol #2011-17 and Continuation of Protocol #2011-17). The study was designed in accordance with the Guidelines for Strengthening the Reporting of Observational Studies in Epidemiology (STROBE) for reporting cross-sectional studies^20^ .

### Definitions of independent variables

Baseline fasting blood glucose (FBG), glycated hemoglobin and triglycerides (TG) were obtained when participants provided blood samples. Body weight, height, and WC were measured during physical examinations at a mobile health center. All these variables were treated as continuous variables^15^.

The calculation formula for TyG, BMI, WHtR, TyG-WC, TyG-BMI, TyG-WHtR, VAI and LAP were provided in Table S1. Participants were divided into four quartiles (Q1, Q2, Q3, Q4) for each measure, with Q1 as the reference group^21^.

### Assessment of OA

Self-reported arthritis is frequently utilized for case definition in epidemiological research^22^. March^23^ et al. indicates an 81% concordance between self-reported OA and clinically diagnosed OA, demonstrating that self-reporting of OA is generally reliable. Participants who answered “Yes” to the question “Has a doctor or other health professional ever told you that you had arthritis?” were classified as having arthritis. These participants were further queried “What type of arthritis was it?” Those who selected the option of “Osteoarthritis” (2003–2010) or “Osteoarthritis or degenerative arthritis” (2011–2016) were diagnosed with OA^19,24^.

### Assessment of covariates

To assess potential confounding factors, covariates selected based on prior research and clinical rationale included age, gender, race, education level, poverty income ratio (PIR), lifestyle factors (drinking and smoking), and chronic diseases (hypertension and diabetes)^25,26^.

Age (years) was considered as a continuous variable. Gender was classified as male or female. Race was divided into five groups of Non-Hispanic White, Non-Hispanic Black, Mexican American, other races and other Hispanic. Education was divided into three categories: < High School, high school, and > High School. The PIR was calculated by dividing household (or individual) income by the poverty threshold for the survey year, and was categorized into three groups: <1.3, 1.3–3.49, and ≥ 3.5. Smoking status was classified as current smoker, former smoker, and never smoker based on responses to the questions: “Have you smoked at least 100 cigarettes in your entire life?” and “Do you currently smoke cigarettes?”. Alcohol consumption was defined by the response to the question: “Have you had at least 12 drinks of any type of alcoholic beverage in any one year?” and categorized into two groups: yes or no.

Hypertension was defined as self-reported diagnosis, use of antihypertensive medication, or blood pressure ≥ 140/90 mmHg. Diabetes status was classified as “diabetes”: based on any of the following criteria: self-reported diagnosis, glycosylated hemoglobin (HbA1c) level ≥ 6.5%, FBG level ≥ 7.0 mmol/L, or use of diabetes medications or insulin^27^.

### Statistical analysis

Participants were classified into two groups based on the presence or absence of OA. Continuous variables are represented as mean ± standard deviation (SD) and compared using the Wilcoxon rank-sum test, while categorical variables are shown as counts (n) and percentages (%) and compared with the chi-square test. Associations between surrogate IR indexes (TyG, TyG-WC, TyG-BMI, TyG-WHtR, VAI, LAP) and OA were evaluated using as categorical form.

We used three logistic regression models to explore the associations between surrogate IR indexes and OA. In Model 1, no covariates were adjusted; Model 2 adjusted for age and gender; Model 3 further adjusted for race, education level, PIR, drinking, smoking, hypertension, and diabetes. Missing covariate values were imputed using multiple imputation. Results are presented as odds ratios (OR) with 95% confidence intervals (CI). Trend analysis treated quartiles as a continuous variable. Restricted cubic spline (RCS) analysis assessed linear and nonlinear dose-response relationships between surrogate IR indexes and OA, with four knots at the 5th, 35th, 65th, and 95th percentiles. Receiver operating characteristic (ROC) curves evaluated diagnostic efficacy, with specificity on the X-axis and sensitivity on the Y-axis, and the area under the curve (AUC) indicating prediction accuracy. Stratification and interaction analyses by gender, age (< 60 years and ≥ 60 years), and smoking status (current smoker, former smoker, and never smoked) examined the effects of TyG-WHtR on OA. In sensitivity analysis, participants with missing covariates, those using lipid-lowering therapy, and individuals with current cancer or pregnancy were excluded. Multiple logistic regression models were then employed to evaluate the association between TyG-WHtR and OA. To ensure representativeness of the American adult population, we followed the weighting guidelines from the National Center for Health Statistics (NCHS) and applied the “fasting sub-sample mobile examination center weight” for weighting^28^. Statistical analyses were conducted using R software (version 4.3.0), with significance defined as *p* < 0.05 (two-sided).

## Results

### Baseline characteristics of participants

This study included 14,715 participants (mean age 45.9 ± 16.5 years), comprising 7205 males (49%) and 7,510 females (51%), representing 194,760,632 American adults, 11% of whom have OA. Table 1 details the demographic and baseline characteristics of OA versus non-OA groups. Increased OA prevalence was observed in higher quartiles of TyG-WC, TyG-BMI, TyG-WHtR, VAI and LAP (all *p* < 0.001), a trend not observed in the non-OA population.

**Table 1:** Baseline characteristics of study participants.

Characteristic	Overall *N* = 14,715 (100%)	non-OA *N* = 13,136 (89%)	OA *N* = 1579 (11%)	*P* value
Age (years)	45.9 ± 16.5	43.9 ± 15.9	60.9 ± 13.0	< 0.001
Gender				< 0.001
Male	7205 (49%)	6635 (51%)	570 (36%)	
Female	7510 (51%)	6501 (49%)	1009 (64%)	
Race				< 0.001
Non-Hispanic White	6544 (68%)	5519 (66%)	1025 (83%)	
Non-Hispanic Black	2860 (11%)	2639 (12%)	221 (6.5%)	
Mexican American	2500 (8.6%)	2369 (9.4%)	131 (3.0%)	
Other Races	1431 (7.2%)	1343 (7.5%)	88 (4.8%)	
Other Hispanic	1380 (5.3%)	1266 (5.7%)	114 (2.5%)	
Education level				0.2
< High school	3738 (17%)	3382 (17%)	356 (16%)	
High School	3304 (22%)	2960 (23%)	344 (21%)	
> High school	7673 (61%)	6794 (61%)	879 (64%)	
PIR				0.001
< 1.3	4654 (21%)	4250 (22%)	404 (17%)	
1.3–3.49	5573 (37%)	4937 (37%)	636 (39%)	
> = 3.5	4488 (42%)	3949 (41%)	539 (45%)	
Smoke status				< 0.001
Current smoker	3064 (21%)	2787 (21%)	277 (18%)	
Former smoker	3549 (24%)	2996 (23%)	553 (35%)	
Never smoker	8102 (55%)	7353 (56%)	749 (47%)	
Alcohol status	10,508 (76%)	9421 (77%)	1087 (74%)	0.067
Diabetes	2399 (12%)	2003 (11%)	396 (21%)	< 0.001
Hypertension	5726 (34%)	4691 (31%)	1035 (60%)	< 0.001
BMI (kg/m^2^**)**	29 ± 7	28 ± 7	31 ± 7	< 0.001
WC (cm)	98 ± 16	98 ± 16	104 ± 16	< 0.001
WHtR	0.58 ± 0.10	0.58 ± 0.09	0.63 ± 0.10	< 0.001
TyG	8.60 ± 0.66	8.58 ± 0.66	8.75 ± 0.64	< 0.001
TyG-WC				< 0.001
Q1	3679 (27%)	3465 (29%)	214 (15%)	
Q2	3679 (25%)	3331 (25%)	348 (23%)	
Q3	3678 (24%)	3226 (24%)	452 (26%)	
Q4	3679 (25%)	3114 (23%)	565 (37%)	
TyG-BMI				< 0.001
Q1	3679 (27%)	3434 (28%)	245 (17%)	
Q2	3679 (25%)	3312 (25%)	367 (23%)	
Q3	3678 (24%)	3270 (24%)	408 (24%)	
Q4	3679 (24%)	3120 (23%)	559 (36%)	
TyG-WHtR				< 0.001
Q1	3679 (28%)	3497 (30%)	182 (13%)	
Q2	3679 (26%)	3337 (26%)	342 (23%)	
Q3	3678 (24%)	3232 (23%)	446 (26%)	
Q4	3679 (23%)	3070 (21%)	609 (37%)	
VAI				< 0.001
Q1	3679 (26%)	3361 (26%)	318 (21%)	
Q2	3679 (26%)	3316 (26%)	363 (22%)	
Q3	3678 (25%)	3263 (24%)	415 (25%)	
Q4	3679 (24%)	3196 (23%)	483 (32%)	
LAP				< 0.001
Q1	3679 (27%)	3476 (28%)	203 (14%)	
Q2	3679 (25%)	3299 (25%)	380 (23%)	
Q3	3678 (24%)	3215 (24%)	463 (27%)	
Q4	3679 (24%)	3146 (23%)	533 (36%)	

*OA* Osteoarthritis, *PIR* poverty income ratio, *BMI* body mass index, *WC* waist circumferences,* WHtR* waist-to-height-ratio, *TyG* triglyceride-glucose index, *VAI* the visceral adiposity index, *LAP* lipid accumulation product.

### Association between surrogate IR indexes and OA

Three logistic regression models were constructed to evaluate the associations between surrogate IR indices and OA (Table 2). In the unadjusted model (Model 1), all indices—TyG, TyG-WC, TyG-BMI, TyG-WHtR, VAI and LAP—were positively associated with OA.

**Table 2:** Associations between surrogate insulin resistance indexes and OA in NHANES 2003–2016.

OA	Model 1		Model 2		Model 3	
	OR (95% CI)	*P* value	OR (95% CI)	*P* value	OR (95% CI)	*P* value
TyG						
Q1	Ref.		Ref.		Ref.	
Q2	1.3318(1.0696, 1.6584)	0.011	1.0203 (0.8145, 1.2780)	0.860	0.9984 (0.7876, 1.2657)	0.989
Q3	1.7792 (1.4452, 2.1905)	< 0.001	1.2448 (0.9981, 1.5525)	0.052	1.2083 (0.9610, 1.5192)	0.104
Q4	2.0540 (1.6297, 2.5889)	< 0.001	1.3438 (1.0402, 1.7360)	0.024	1.2253 (0.9275, 1.6187)	0.151
*P* for trend	< 0.001		0.004		0.046	
TyG-WC						
Q1	Ref.		Ref.		Ref.	
Q2	1.7893(1.4686, 2.1800)	< 0.001	1.3077 (1.0527, 1.6246)	0.016	1.3415 (1.0653, 1.6893)	0.013
Q3	2.1754 (1.7851, 2.6512)	< 0.001	1.5359 (1.2269, 1.9226)	< 0.001	1.5793 (1.2464, 2.0012)	< 0.001
Q4	3.1660 (2.5571, 3.9198)	< 0.001	2.3830 (1.9105, 2.9725)	< 0.001	2.3475 (1.8388, 2.9971)	< 0.001
*P* for trend	< 0.001		< 0.001		< 0.001	
TyG-BMI						
Q1	Ref.		Ref.		Ref.	
Q2	1.5503(1.2263, 1.9600)	< 0.001	1.2116 (0.9467, 1.5506)	0.126	1.2621 (0.9793, 1.6265)	0.072
Q3	1.6813 (1.3996, 2.0197)	< 0.001	1.3624 (1.1103, 1.6718)	0.003	1.4468 (1.1637, 1.7987)	0.001
Q4	2.6510 (2.1435, 3.2787)	< 0.001	2.3825 (1.9140, 2.9657)	< 0.001	2.4969 (1.9808, 3.1474)	< 0.001
*P* for trend	< 0.001		< 0.001		< 0.001	
TyG-WHtR						
Q1	Ref.		Ref.		Ref.	
Q2	2.0234(1.6705, 2.4509)	< 0.001	1.2869 (1.0383, 1.5950)	0.022	1.3036 (1.0439, 1.6280)	0.020
Q3	2.5759 (2.1177, 3.1334)	< 0.001	1.4970 (1.2120, 1.8489)	< 0.001	1.5825 (1.2587, 1.9894)	< 0.001
Q4	4.0636 (3.3238, 4.9679)	< 0.001	2.1933 (1.7535, 2.7433)	< 0.001	2.2444 (1.7604, 2.8615)	< 0.001
*P* for trend	< 0.001		< 0.001		< 0.001	
VAI						
Q1	Ref.		Ref.		Ref.	
Q2	1.0333 (0.8368, 1.2759)	0.759	0.8968 (0.7229, 1.1126)	0.319	0.8802 (0.7069, 1.0961)	0.251
Q3	1.2674 (1.0621, 1.5125)	0.009	1.0029 (0.8379, 1.2005)	0.974	0.9773 (0.8098, 1.1793)	0.809
Q4	1.7325 (1.4268, 2.1037)	< 0.001	1.3677 (1.1111, 1.6836)	0.003	1.2597 (1.0103, 1.5708)	0.040
*P* for trend	< 0.001		0.002		0.016	
LAP						
Q1	Ref.		Ref.		Ref.	
Q2	1.8001 (1.4373, 2.2543)	< 0.001	1.2246 (0.9700, 1.5460)	0.088	1.2126 (0.9574, 1.5359)	0.109
Q3	2.3063 (1.8488, 2.8772)	< 0.001	1.4184 (1.1245, 1.7890)	0.004	1.3925 (1.0964, 1.7685)	0.007
Q4	3.1026 (2.4690, 3.8988)	< 0.001	1.9879 (1.5776, 2.5049)	< 0.001	1.8395 (1.4515, 2.3312)	< 0.001
*P* for trend	< 0.001		< 0.001		< 0.001	

*OA* Osteoarthritis, *BMI* body mass index, *WC* waist circumferences, *WHtR* waist-to-height-ratio, *TyG* triglyceride-glucose index, *VAI* the visceral adiposity index, *LAP* lipid accumulation product, *OR* Odds Ratio, *CI* Confidence interva, *Q* Quartile, *Ref* Reference. Model 1 (unadjusted) did not adjust for any covariates. Model 2 adjustments were made for sex and age. Model 3 further adjustments for race, education level, poverty income ratio (PIR), drinking and smoking status, hypertension and diabetes. VAI and LAP did not need adjust for sex.

This association remained after adjusting for age and sex (Model 2). After further adjusting for race, education, PIR, alcohol use, smoking status, hypertension, and diabetes (Model 3), TyG-WC, TyG-BMI, TyG-WHtR and LAP remained significantly associated with increased the prevalence of OA (all *p* < 0.01). When dividing TyG-WC, TyG-BMI, TyG-WHtR and LAP into quartiles, participants in the highest quartile exhibited a higher prevalence of OA compared to those in the lowest quartile, with ORs [95% CI] for Q4 of 2.3475 [1.8388, 2.9971], 2.4969 [1.9808, 3.1474], 2.2444 [1.7604, 2.8615] and 1.8395 [1.4515, 2.3312]. All *p* for trend were significant.

**Fig. 2:**
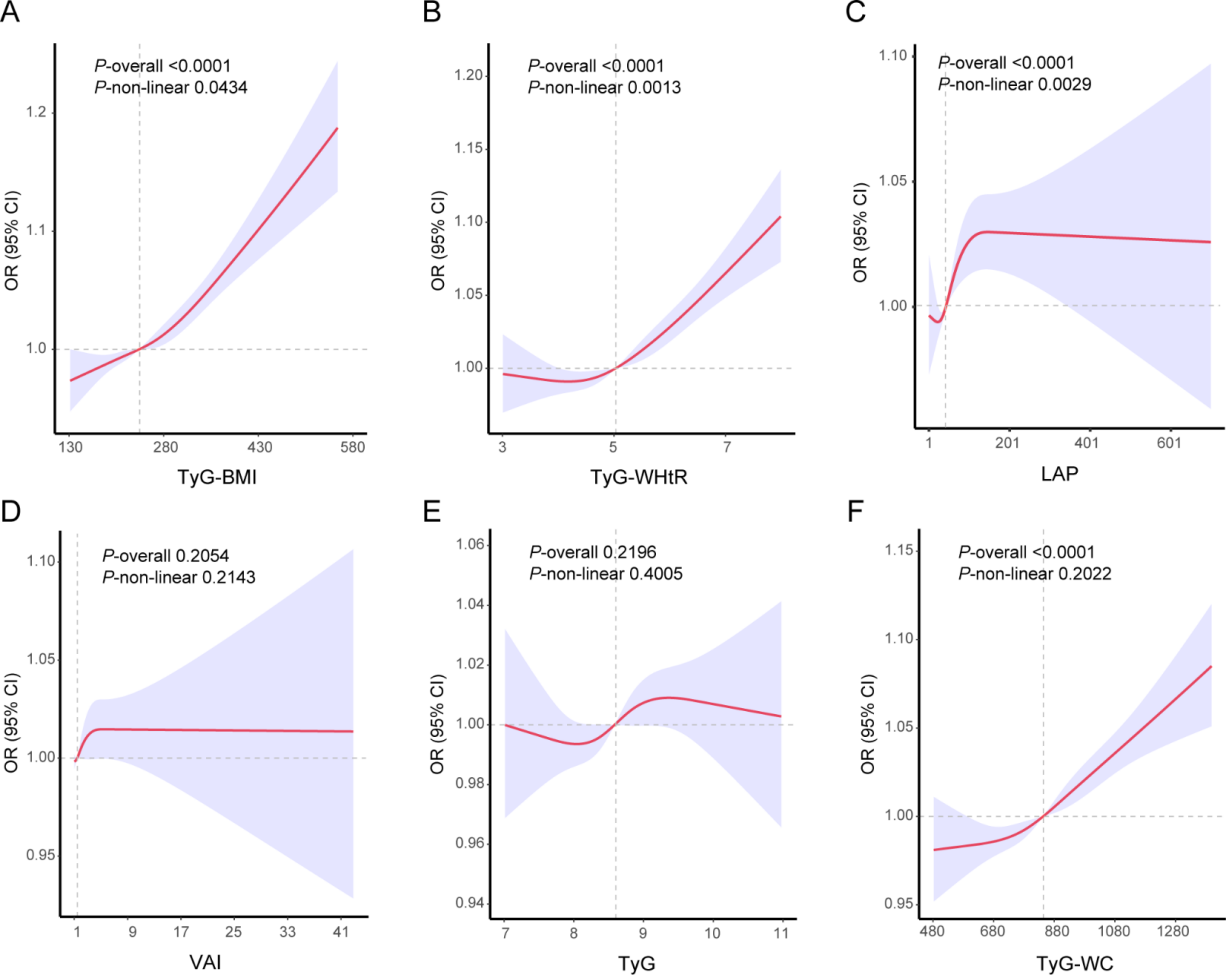
Restricted cubic spline plot model. The adjusted restricted cubic spline plot model shows an association between surrogate insulin resistance indexes and OA among all participants. The model was adjusted for gender, age, race, education level, PIR, drinking and smoking status, hypertension and diabetes (VAI and LAP did not need adjust for gender). The red solid line and the purple shaded area represent the estimated odds ratio and its 95% confidence interval, respectively.

Additionally, we used RCS to evaluate the associations of TyG, TyG-BMI, TyG-WC, TyG-WHtR, VAI and LAP with OA. After adjusting for all confounders in model 3, only the associations of TyG-BMI (*p*-overall < 0.0001, *p*-non-linear = 0.0434), TyG-WHtR (*p*-overall < 0.0001, *p*-non-linear = 0.0013) and LAP (*p*-overall < 0.0001, *p*-non-linear = 0.0029) displayed a nonlinear relationship with OA incidence (Fig. 2).

### Diagnostic efficacy of surrogate IR indexes for OA

**Fig. 3:**
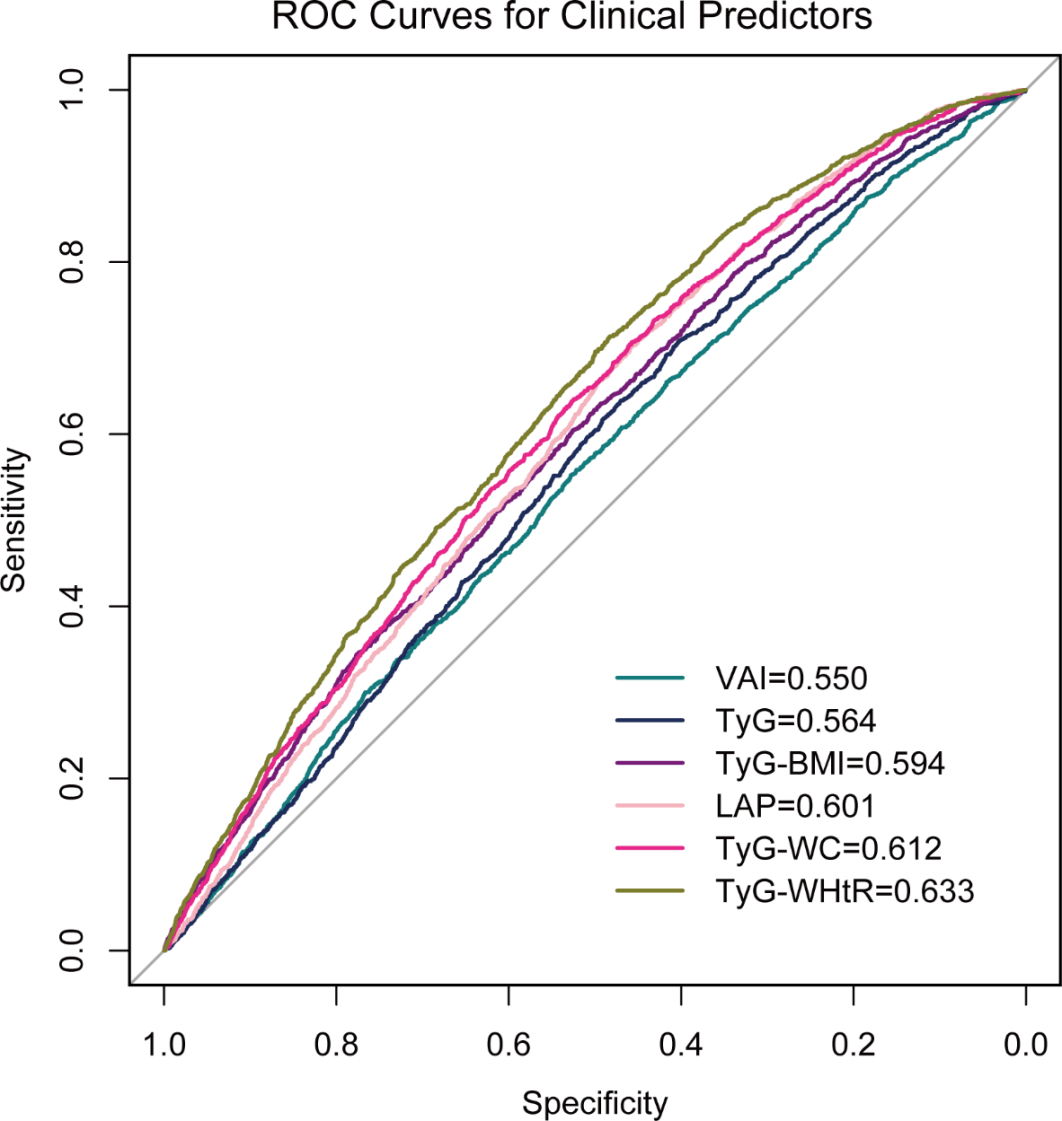
The ROC curves of surrogate indexes for predicting OA.

To further investigate the diagnostic efficacy of these indices for OA, we performed ROC curve analysis. The results indicated that TyG-WHtR had the highest diagnostic efficacy (AUC 0.633), suggesting that TyG-WHtR may be a more suitable indicator for assessing OA (Fig. 3).

### Stratified and sensitivity analyses of potential effect modifiers

**Fig. 4:**
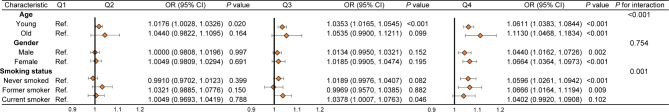
Associations between TyG-WHtR and OA in different subgroups. Analysis of age was adjusted for gender, race, education level, PIR, drinking and smoking status, hypertension and diabetes. Analysis of gender was adjusted for age, race, education level, PIR, drinking and smoking status, hypertension and diabetes. Analysis of smoking status was adjusted for gender, age, race, education level, PIR, drinking status, hypertension and diabetes. *OR* odds ratio, *CI* Confidence Interval, *Ref* Reference, *Q* Quartile.

To further assess the association between TyG-WHtR and OA, we stratified the population by age, sex, and smoking status and conducted interactive analyses. A significant interaction was observed between TyG-WHtR and both age (*p* < 0.001) and smoking status (*p* = 0.001), while no interaction was found with sex (*p* = 0.754). After fully adjusting for confounders such as sex, race, education level, PIR, alcohol consumption, smoking status, hypertension, and diabetes, TyG-WHtR was positively associated with OA in both older (≥ 60 years) and younger (< 60 years) populations. When divided into quartiles, the ORs [95% CIs] for the younger population were: Q2: 1.0176 [1.0028, 1.0326], *p* = 0.020; Q3: 1.0353 [1.0165, 1.0545], *p* < 0.001; and Q4: 1.0611 [1.0383, 1.0844], *p* < 0.001. In the older population, higher levels of TyG-WHtR also significantly impacted the development of OA, with an OR [95% CI] for Q4 of 1.1130 [1.0468, 1.1834], *p* < 0.001. The results of the study stratified by sex indicated that higher TyG-WHtR levels significantly affected the incidence of OA in both males and females, demonstrating a positive correlation with ORs [95% CIs] for Q4: 1.0440 [1.0162, 1.0726] in males (*p* = 0.002) and 1.0664 [1.0364, 1.0973] in females (*p* < 0.001). Subgroup analyses based on smoking status indicated that TyG-WHtR significantly impacted OA development in both nonsmoking and former smoking populations, particularly at higher TyG-WHtR levels. The ORs [95% CIs] for Q4 were 1.0596 [1.0261, 1.0942] in the nonsmoking population (*p* < 0.001) and 1.0666 [1.0164, 1.1194] in the former smoking population (*p* = 0.009) (Fig. 4).

To assess the robustness of the association between IR and OA, we accounted for potential confounders including lipid-lowering therapy, cancer, and pregnancy. Sensitivity analyses were conducted by excluding participants with missing covariate data (*n* = 1997), those on lipid-lowering therapy (*n* = 2482), and those with a current diagnosis of cancer or pregnancy (*n* = 1532). Further analysis of the association between TyG-WHtR and OA revealed that TyG-WHtR remained significantly associated with OA risk after full adjustment for confounding factors (all *p* < 0.001), indicating a robust relationship between TyG-WHtR and OA (Table S2).

## Discussion

OA accounts for a significant proportion of the population, is one of the leading causes of disability among the elderly, and represents a considerable social burden. Therefore, identifying potential risk and prognostic factors could lead to cost savings in the management of this population^29^. IR and obesity are critical factors influencing the development of OA^7^, with the TyG serving as a reliable measure for assessing IR^9,12,30^. This index relies exclusively on the patient’s TG and FBG levels, making it highly accessible and cost-effective. Furthermore, TyG has been associated with the prevalence of OA^11^, diabetes mellitus^31^, cardiovascular disease^15^, and hypertension^32^. Recent studies have indicated that novel TyG-related indexes such as TyG-WC, TyG-BMI, and TyG-WHtR exhibit superior predictive abilities compared to TyG alone regarding diseases, including pre-diabetes and cardiovascular conditions^13,16,33^. While previous research has shown that the VAI, LAP effectively assess IR status^33^, which is linked to diabetes^34^, stroke^35^, atherosclerosis^36^, and coronary artery disease^33^, the relationships between these surrogate IR indexes and the prevalence of OA, as well as their advantages for OA diagnostic efficacy, remain unclear.

This study aims to explore the associations between the VAI, LAP, and TyG combinations with obesity indicators, concerning the prevalence of OA. Additionally, it compares the diagnostic efficacy of surrogate IR indices. These results are consistent with earlier studies. Zhang^19^ et al.. found that TyG-BMI and TyG-WHtR were positively associated with arthritis prevalence in both Chinese and American populations. Furthermore, these indices demonstrated stronger correlations with arthritis diagnoses compared to TyG. Liu^33^ et al. demonstrated that TyG, TyG-WC, TyG-BMI, TyG-WHtR and LAP were independently associated with an increased prevalence of coronary heart disease. Notably, TyG-WHtR and TyG-BMI exhibited significant predictive capacities. Additionally, Liu^36^ et al. reported that Chinese visceral adiposity index, TyG-WC, TyG-WHtR, and LAP were independently associated with an increased prevalence of carotid atherosclerosis, with Chinese visceral adiposity index and TyG-WHtR emerging as the most suitable predictive markers.

Another finding of this study is that the associations between surrogate IR indexes, particularly TyG-WHtR and OA vary across populations with differing characteristics. Specifically, the associations of TyG-WHtR with OA are more pronounced among younger individuals. The results of a previous study by Puenpatom^37^ et al. indicated an increased prevalence of MetS and OA among younger American adults, with this association diminishing with age, which parallels our findings. TyG-WHtR significantly correlated with OA incidence in both sexes, suggesting that there is no sex difference in the increased prevalence of OA due to insulin resistance and obesity. Controversy persists regarding the relationship between smoking and OA. Numerous clinical studies across diverse populations have reported a negative association between smoking and OA incidence^38–41^. In contrast, large national studies in the United States reveal a positive correlation between smoking and the prevalence of adult OA^42^. While the findings from the Osteoarthritis Initiative (OAI) in the American and the Tasmanian Older Adult Cohort (TASOAC) in Australia did not demonstrate a significant association between smoking and OA incidence^43^, a trend that is echoed in cross-sectional studies from Korea^44^ and Denmark^45^. The present study identified a positive association between TyG-WHtR and the prevalence of OA in non-smokers, a similar association among former smokers. Previous studies have shown that inflammatory markers are significantly elevated in smokers compared to non-smokers. Cigarette smoke promotes the release of inflammatory cytokines, such as tumor necrosis factor-ɑ (TNF-ɑ), interleukin-6 (IL-6) and high-sensitivity C-reactive protein (hsCRP), which plays a crucial role in the progression of OA^19,46,47^. Moreover, nicotine impairs osteoblast function, leading to tissue hypoxia, which subsequently activates osteoclasts and promotes bone resorption^48^. On the other hand, obesity remains a major risk factor for OA, while smokers tend to be thinner than non-smokers and generally have a lower risk of OA. This may explain the lack of statistically significant differences observed in the evaluation of OA incidence among smokers using the TyG-WHtR. These findings indicate that, regardless of nonsmoking or former smoking, actively improving IR and managing obesity can significantly decrease the likelihood of OA development.

Insulin is a crucial regulator of synovial inflammation and catabolism, and individuals with IR frequently exhibit MetS, such as obesity. IR reduces the body’s capacity to suppress the production of inflammatory mediators, thereby increasing the risk of OA, particularly in obese individuals^49^. Obesity impacts cartilage tissue not only by imposing excessive mechanical load but also by inducing inflammatory responses associated with adipose tissue. In individuals who are overweight or obese, adipose deposition results in elevated plasma levels of TNF-ɑ and IL-6. TNF-ɑ inhibits the tyrosine phosphorylation of insulin receptors and insulin receptor substrate 1 (IRS-1) kinases while promoting the serine phosphorylation of IRS-1, leading to dysfunction in adipocytes and skeletal muscle cells, which in turn contributes to IR in both adipose and non-adipose tissues. The development of IR prompts the pancreas to produce additional insulin, further enhancing fat synthesis and storage. Obesity influences the development of OA not only by directly impacting cartilage tissue through excessive mechanical loading but also by inducing inflammatory responses associated with adipose tissue^50^. In individuals who are overweight or obese, adipose deposition leads to increased plasma levels of TNF-ɑ and IL-6^51^. TNF-ɑ inhibits the tyrosine phosphorylation of insulin receptors and IRS-1 kinases while promoting serine phosphorylation of IRS-1, resulting in dysfunction of adipocytes and skeletal muscle cells, which in turn leads to IR across both adipose and non-adipose tissues^52^. The onset of IR prompts the pancreas to produce additional insulin, further enhancing fat synthesis and storage^53^. Furthermore, preclinical and clinical findings indicate that MetS and obesity promote the polarization of macrophages from a reparative anti-inflammatory M2 phenotype to a pro-inflammatory M1 phenotype, leading to the secretion of substantial quantities of cytokines and mediators, including TNF-ɑ, IL-6, interleukin-2, and interleukin-1, which exacerbate synovitis and cartilage degradation^54–56^. The vicious cycle between IR and obesity further contributes to the development of OA.

The present study had the following strengths. First, we investigate the association between IR and OA using multiple surrogate IR indexes, while identifying the most suitable indicators for assessing OA. This study yielded largely consistent results across various subgroups and sensitivity analyses, further validating our findings. Second, by considering the impact of the vicious cycle between IR and obesity on OA development, the surrogate IR indexes effectively integrate responses from both IR and obesity to enhance OA assessment. However, this study also had some limitations. Firstly, the reliance on personal interviews to diagnose OA without relevant laboratory data, such as anti-cyclic citrullinated peptide antibodies and rheumatoid factor, may introduce inaccuracies in data collection, despite the reported high concordance between self-reported and clinically defined OA. Secondly, the cross-sectional study design prevents the establishment of causal relationships between the studied variables, necessitating further prospective studies and intervention trials to acquire more robust evidence.

## Conclusion

In conclusion, this cross-sectional NHANES study (2003–2016) indicates that surrogate IR indexes are positively associated with OA. Combining multiple indices display superior diagnostic relevance compared to TyG, with TyG-WHtR showing the highest diagnostic efficacy.

## Electronic supplementary material

Below is the link to the electronic supplementary material.

Supplementary Material 1

## Data Availability

Data are publicly available at https://www.cdc.gov/nchs/nhanes.

## Abbreviations

OA: Osteoarthritis
NHANES: National Health and Nutrition Examination Survey
MetS: Metabolic syndrome
IR: Insulin resistance
VAI: Visceral adiposity index
LAP: Lipid accumulation product
TyG: Triglyceride-glucose index
TyG-WC: Glucose triglyceride-waist circumference
TyG-BMI: Glucose triglyceride-body mass index
TyG-WHtR: Glucose triglyceride-waist height ratio
BMI: Body mass index
WHtR: Waist height ratio
WC: Waist circumference
FBG: Fasting blood glucose
TG: Triglycerides
PIR: Poverty income ratio
SD: Standard deviation
OR: Odds ratio
CI: Confidence intervals
RCS: Restricted cubic spline
ROC: Receiver operating characteristic
AUC: Area under the curve
NCHS: National Center for Health Statistics
TNF-ɑ: Tumor necrosis factor-ɑ
IL-6: Interleukin-6
IRS-1: Insulin receptor substrate 1
